# Supplementary material for: Induction of lysosomal exocytosis and biogenesis via TRPML1 activation for the treatment of uranium-induced nephrotoxicity
Source: Nat Commun. 2023 Jul 6;14:3997. doi: 10.1038/s41467-023-39716-7 (PMC10326073; doi:10.1038/s41467-023-39716-7)
Supplement: Supplementary file 1 — Supplementary Information [file 41467_2023_39716_MOESM1_ESM.pdf]

Induction of lysosomal exocytosis and biogenesis via TRPML1 activation for the treatment of uranium-induced nephrotoxicity

Dengqin Zhong[1,2], Ruiyun Wang[1,2], Hongjing Zhang[1,2], Mengmeng Wang[1], Xuxia Zhang[1], Honghong Chen[1*]

[1] Institute of Radiation Medicine, Shanghai Medical College, Fudan University, Shanghai, P. R. China

[2] These authors contributed equally: Dengqin Zhong, Ruiyun Wang, Hongjing Zhang.

[*] Corresponding author

Supplementary Information

Supplementary Figures 1-11

Supplementary Table 1

Supplementary Materials and Methods

Supplementary References

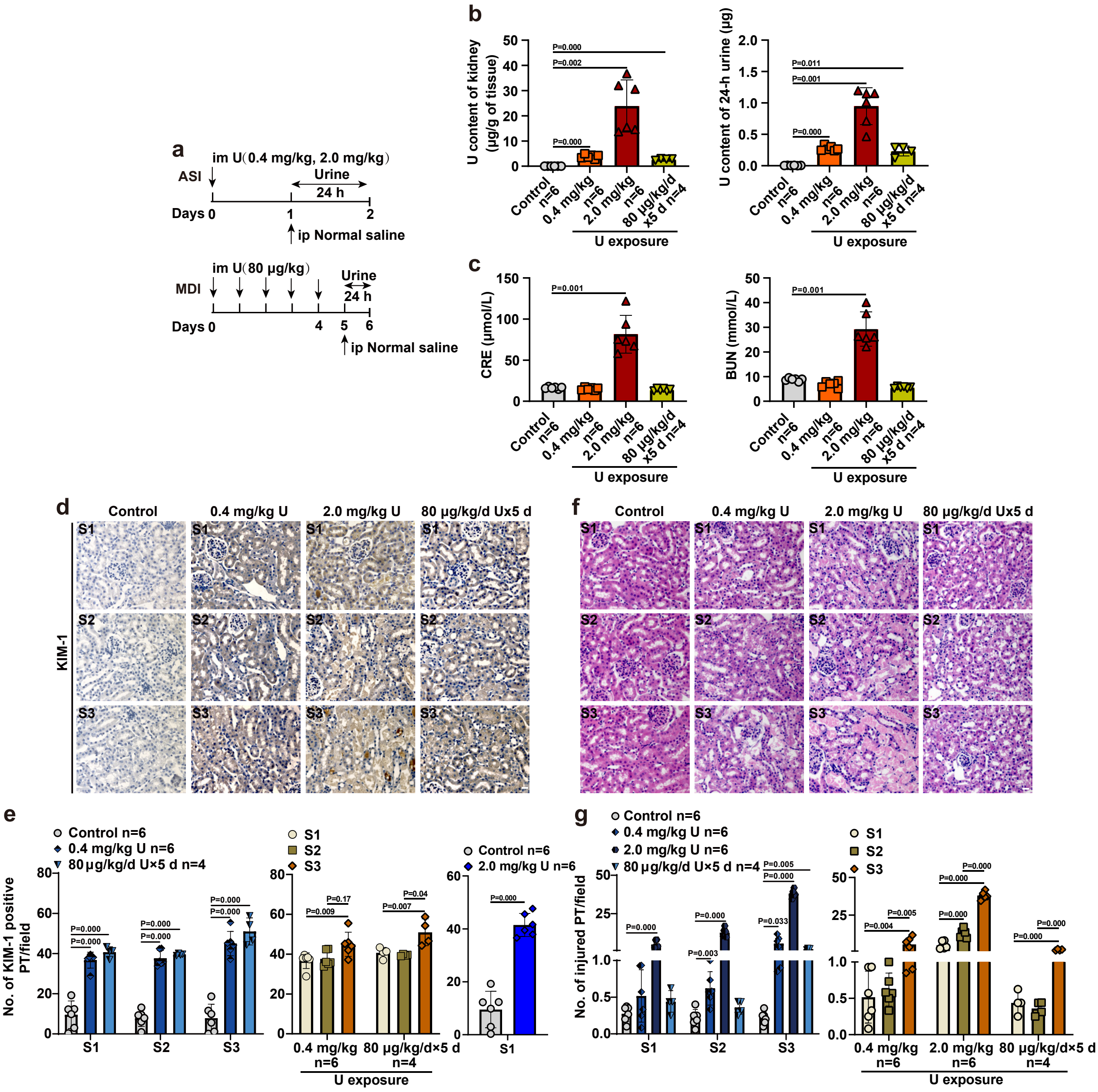
a
ASI
im U(0.4 mg/kg, 2.0 mg/kg)
Urine
24 h
Days 0
1
2
ip Normal saline
MDI
im U(80 μg/kg)
Urine
24 h
Days 0
4
5
6
ip Normal saline
b
U content of kidney (μg/g of tissue)
U content of 24-h urine (μg)
Control n=6
0.4 mg/kg n=6
2.0 mg/kg n=6
80 μg/kg/d x5 d n=4
U exposure
c
CRE (μmol/L)
BUN (mmol/L)
d
Control
0.4 mg/kg U
2.0 mg/kg U
80 μg/kg/d Ux5 d
KIM-1
S1
S2
S3
f
e
Control n=6
0.4 mg/kg U n=6
80 μg/kg/d U×5 d n=4
No. of KIM-1 positive PT/field
2.0 mg/kg U n=6
0.4 mg/kg n=6
80 μg/kg/d×5 d n=4
g
No. of injured PT/field
2.0 mg/kg n=6

Supplementary Figure 1. Single- or multiple-dose exposure to U lead to the U accumulation and proximal tubule injuries in mouse kidney. (**a**) Schematic representation of the experiments on single- (0.4 or 2.0 mg/kg) or multiple-dose (80 μg/kg/day for 5 days) U exposure to mimic acute or chronic U exposure in BALB/c male mice. *n* = 6 mice for control group and single-dose U exposure groups. *n* = 4 mice for multiple-dose U exposure group. (**b**) U contents in kidney tissues and 24-h urine samples of mice before euthanasia after single- or multiple-dose U exposure. (**c**) CRE and BUN levels after single- or multiple-dose U exposure. (**d**) Representative images of immunohistochemical staining of KIM-1 in the S1, S2 and S3 segments of proximal tubules in renal cortex of mice after single- or multiple-dose U exposure. (**e**) Quantitative analysis of KIM-1 levels in the S1, S2 and S3 segments of proximal tubules as shown in **d**. (**f**) Representative images of H&E staining of the S1, S2 and S3 segments of proximal tubules in renal cortex of mice after single- or multiple-dose U exposure. (**g**) Quantitative analysis of pathological injury of proximal tubules with necrotic or exfoliated cells in the S1, S2 and S3 segments of proximal tubules as shown in **f**. im: intramuscular injection; ip: intraperitoneal injection; ASI: a single injection; MDI: multiple dose injection; PT: proximal tubule. Data represent mean ± SD. Statistical significance was evaluated by one-way ANOVA with LSD's post hoc test (**b**, **c**, the first and second graphs in **e**, **g**) or two-tailed Student's t-test (the third graph in **e**). Source data are provided as a Source Data file. All the images share the same scale bar (20 μm).

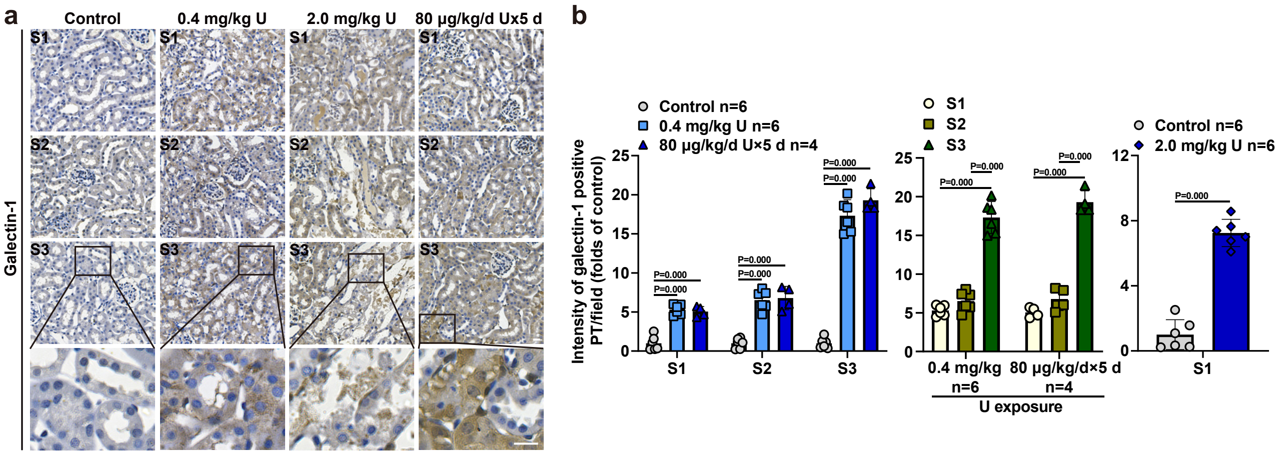

a
Control
0.4 mg/kg U
2.0 mg/kg U
80 µg/kg/d Ux5 d
S1
S2
S3
Galectin-1
b
Control n=6
0.4 mg/kg U n=6
80 µg/kg/d U×5 d n=4
Intensity of galectin-1 positive PT/field (folds of control)
P=0.000
S1
S2
S3
0.4 mg/kg n=6
80 µg/kg/d×5 d n=4
U exposure
Control n=6
2.0 mg/kg U n=6

Supplementary Figure 2. Single- or multiple-dose U exposure leads to LMP in PTECs of mouse kidney. The male mice with single- or multiple-dose U exposure were described in **Supplementary Figure 1a**. *n* = 6 mice for control group and single-dose U exposure groups. *n* = 4 mice for multiple-dose U exposure group. (**a**) Representative images of immunohistochemical staining of galectin-1 in the S1, S2 and S3 segments of proximal tubules in renal cortex of mice after single- or multiple-dose exposure to U. Boxed areas are enlarged below. Images share the same scale bar (20 μm). (**b**) Quantitative analysis of galectin-1 staining in the S1, S2 and S3 segments of the proximal tubules as shown in **a**. PT: proximal tubule. Data represent mean ± SD. Statistical significance was evaluated by one-way ANOVA with LSD's post hoc test (the first and second graphs in **b**) or two-tailed Student's t-test (the third graph in **b**). Source data are provided as a Source Data file.

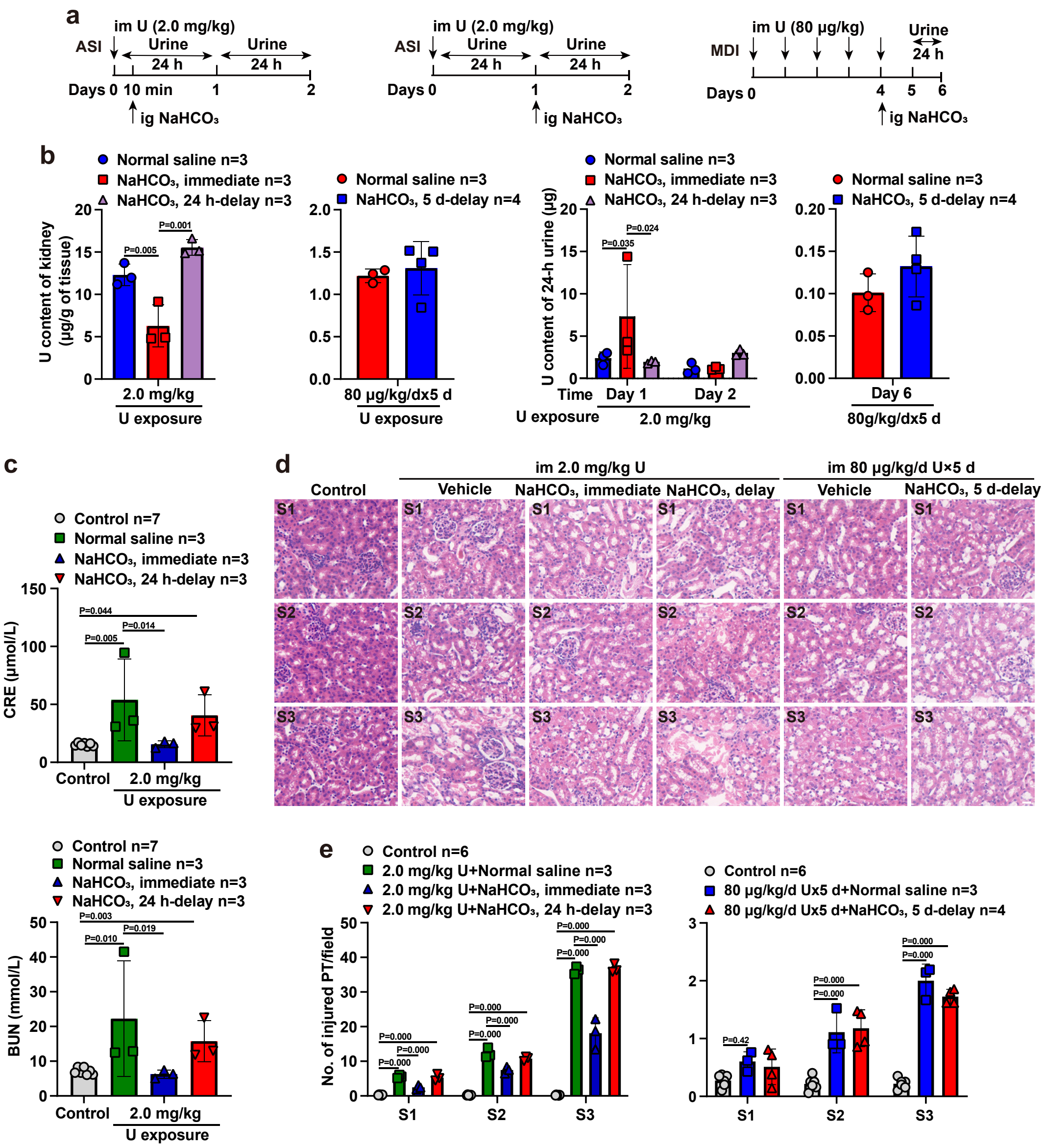

a
im U (2.0 mg/kg)
ASI
Urine
24 h
Urine
24 h
Days 0 10 min 1 2
ig NaHCO₃
im U (2.0 mg/kg)
ASI
Urine
24 h
Urine
24 h
Days 0 1 2
ig NaHCO₃
im U (80 μg/kg)
MDI
Urine
24 h
Days 0 4 5 6
ig NaHCO₃
b
Normal saline n=3
NaHCO₃, immediate n=3
NaHCO₃, 24 h-delay n=3
U content of kidney (μg/g of tissue)
2.0 mg/kg
U exposure
Normal saline n=3
NaHCO₃, 5 d-delay n=4
80 μg/kg/dx5 d
U exposure
U content of 24-h urine (μg)
Time Day 1 Day 2
U exposure 2.0 mg/kg
Day 6
80g/kg/dx5 d
c
Control n=7
Normal saline n=3
NaHCO₃, immediate n=3
NaHCO₃, 24 h-delay n=3
CRE (μmol/L)
Control 2.0 mg/kg
U exposure
d
Control
im 2.0 mg/kg U
Vehicle
NaHCO₃, immediate
NaHCO₃, delay
im 80 μg/kg/d U×5 d
Vehicle
NaHCO₃, 5 d-delay
S1
S2
S3
BUN (mmol/L)
Control 2.0 mg/kg
U exposure
e
Control n=6
2.0 mg/kg U+Normal saline n=3
2.0 mg/kg U+NaHCO₃, immediate n=3
2.0 mg/kg U+NaHCO₃, 24 h-delay n=3
No. of injured PT/field
Control n=6
80 μg/kg/d Ux5 d+Normal saline n=3
80 μg/kg/d Ux5 d+NaHCO₃, 5 d-delay n=4
S1 S2 S3

Supplementary Figure 3. Effect of immediate and delayed administration of sodium bicarbonate on removing U and protecting against U nephrotoxicity after single- or multiple-dose U exposure in mice. (a) Schematic representation of the experiments on single- or multiple-dose U exposure followed by immediate and delayed administration of sodium bicarbonate in BALB/c male mice. **(b)** U contents in kidney tissues and the last 24-h urine from mice with single- or multiple-dose U exposure and immediate and delayed sodium bicarbonate treatment. n = 3 mice for control groups, single-dose U exposure alone group, single-dose U exposure plus immediate sodium bicarbonate treatment group, and single-dose U exposure plus delayed sodium bicarbonate treatment group. n = 4 mice for multiple-dose U exposure plus delayed sodium bicarbonate treatment group. **(c)** The CRE and BUN levels in mice after single-dose U exposure and immediate and delayed treatment with sodium bicarbonate. n = 7 mice for control group, and n = 3 mice for U exposure alone group and U exposure plus sodium bicarbonate treatment groups. **(d)** Representative H&E staining in the S1, S2 and S3 segments of proximal tubules in renal cortex of mice after single-dose U exposure and immediate and delayed treatment with sodium bicarbonate. Images share the same scale bar (20 μm). **(e)** Quantitative analysis of pathological injury of proximal tubules with necrotic or exfoliated cells in the S1, S2 and S3 segments of proximal tubules as shown in **d**. n = 6 mice for control groups, n = 3 mice for single-dose U exposure alone group, multiple-dose-dose U exposure alone group and single-dose U exposure plus sodium bicarbonate treatment groups, n = 4 mice for in multiple-dose U exposure plus sodium bicarbonate treatment group. im: intramuscular injection; ig: intragastric administration; ASI: a single injection; MDI: multiple dose injection; PT: proximal tubule. Data represent mean ± SD. Statistical significance was evaluated by one-way

ANOVA with LSD’s post hoc test (the first and third graphs in **b**, **c**, **e**) or two-tailed Student’s t-test (the second and fourth graphs in **b**). Source data are provided as a Source Data file.

a

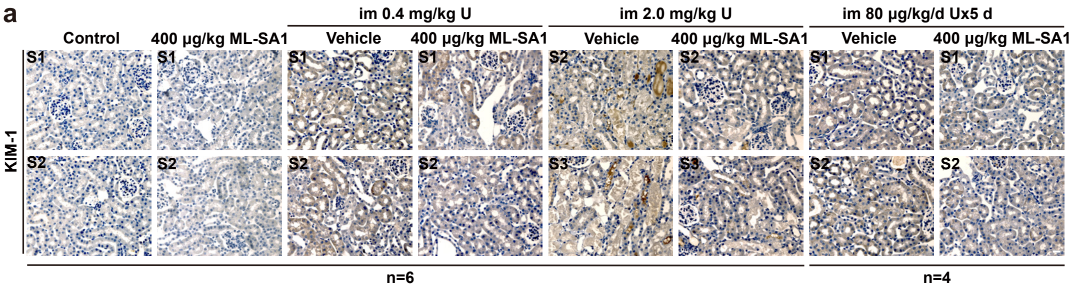
im 0.4 mg/kg U
im 2.0 mg/kg U
im 80 μg/kg/d Ux5 d
Control
400 μg/kg ML-SA1
Vehicle
400 μg/kg ML-SA1
Vehicle
400 μg/kg ML-SA1
Vehicle
400 μg/kg ML-SA1
KIM-1
S1
S2
S3
n=6
n=4

b

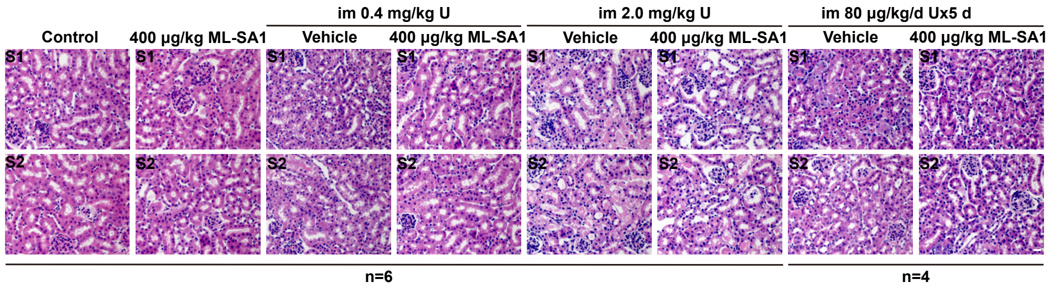
im 0.4 mg/kg U
im 2.0 mg/kg U
im 80 μg/kg/d Ux5 d
Control
400 μg/kg ML-SA1
Vehicle
400 μg/kg ML-SA1
Vehicle
400 μg/kg ML-SA1
Vehicle
400 μg/kg ML-SA1
S1
S2
n=6
n=4

Supplementary Figure 4. TRPML1 agonist ML-SA1 reduces the U-induced renal proximal tubule injury after single- or multiple-dose U exposure in mice. (**a**, **b**) Representative immunohistochemical staining of KIM-1 (**a**) and H&E staining (**b**) in S1 and S2 segments or S2 and S3 segments of proximal tubules in renal cortex of male mice treated with vehicle or ML-SA1 at 400 μg/kg after single- or multiple-dose U exposure. The kidney tissues were harvested from mice after 24 h treatment of ML-SA1 as indicated in the **Fig. 1a** and then examined. $n = 6$ mice for control groups, ML-SA1 treatment alone groups, single-dose U exposure alone groups and single-dose U exposure plus ML-SA1 treatment groups. $n = 4$ mice for multiple-dose U exposure alone group and multiple-dose U exposure plus ML-SA1 treatment group. Images share the same scale bar (20 μm).

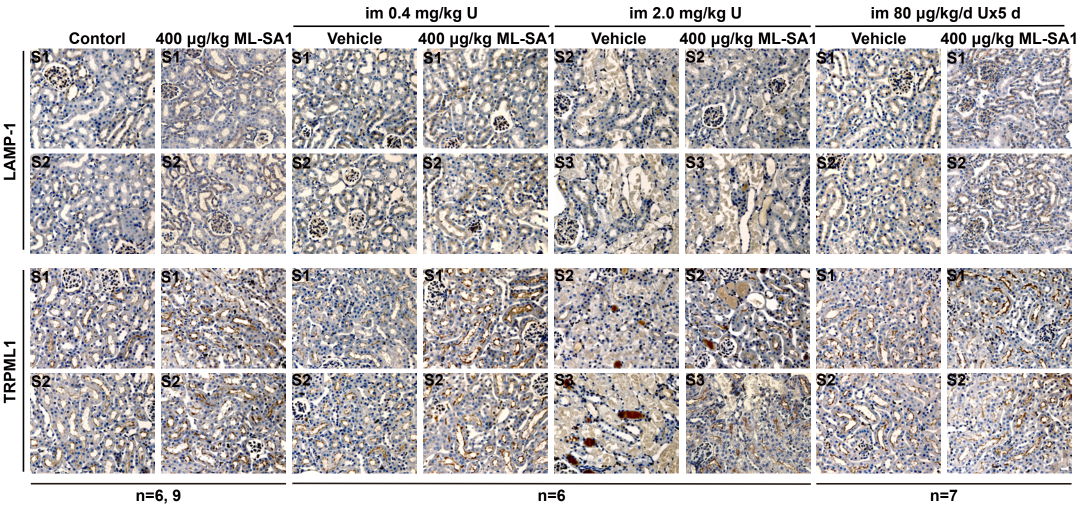
im 0.4 mg/kg U
im 2.0 mg/kg U
im 80 µg/kg/d Ux5 d
Contorl
400 µg/kg ML-SA1
Vehicle
400 µg/kg ML-SA1
Vehicle
400 µg/kg ML-SA1
Vehicle
400 µg/kg ML-SA1
LAMP-1
TRPML1
S1
S2
S3
n=6, 9
n=6
n=7

Supplementary Figure 5. TRPML1 agonist ML-SA1 increases the levels of lysosomal membrane proteins in the apical membrane of renal proximal tubules in mice after single- or multiple-dose U exposure. Representative images of immunohistochemical staining of LAMP-1 and TRPML1 in S1 and S2 segments or S2 and S3 segments of proximal tubules in renal cortex of male mice treated with vehicle or ML-SA1 at 400 μg/kg after single- or multiple-dose U exposure. The kidney tissues were harvested from mice after 24 h treatment of ML-SA1 as indicated in the **Fig. 1a** and then examined. $n = 6$ mice for control group, ML-SA1 treatment alone group, single-dose U exposure alone groups and single-dose U exposure plus ML-SA1 treatment groups. $n = 7$ mice for multiple-dose U exposure alone group and multiple-dose U exposure plus ML-SA1 treatment group, and $n = 9$ mice for corresponding control group and ML-SA1 treatment alone group. Images share the same scale bar (20 μm).

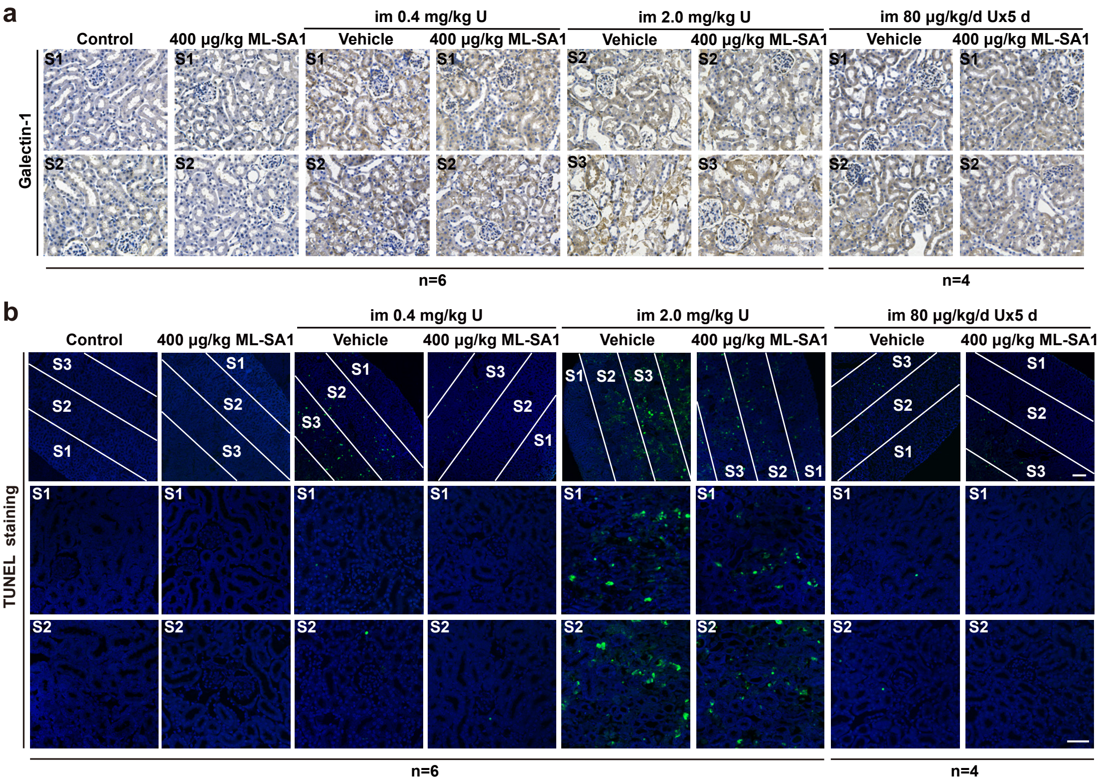
a
Control
400 µg/kg ML-SA1
im 0.4 mg/kg U
Vehicle
400 µg/kg ML-SA1
im 2.0 mg/kg U
Vehicle
400 µg/kg ML-SA1
im 80 µg/kg/d Ux5 d
Vehicle
400 µg/kg ML-SA1
Galectin-1
n=6
n=4
b
Control
400 µg/kg ML-SA1
im 0.4 mg/kg U
Vehicle
400 µg/kg ML-SA1
im 2.0 mg/kg U
Vehicle
400 µg/kg ML-SA1
im 80 µg/kg/d Ux5 d
Vehicle
400 µg/kg ML-SA1
TUNEL staining
n=6
n=4

Supplementary Figure 6. TRPML1 agonist ML-SA1 reduces the U-induced LMP and LMP-related apoptosis of renal PTECs after single- or multiple-dose U exposure in mice. (**a**) Representative images of immunohistochemical staining of galectin-1 in S1 and S2 segments or S2 and S3 segments of proximal tubules in renal cortex of male mice treated with vehicle or ML-SA1 at 400 μg/kg after single- or multiple-dose U exposure. (**b**) Representative images of TUNEL staining in renal cortex and S1 and S2 segments of proximal tubules in renal cortex of mice treated with vehicle or ML-SA1 at 400 μg/kg after single- or multiple-dose U exposure. The kidney tissues were harvested from mice after 24 h treatment of ML-SA1 as indicated in the **Fig. 1a** and then examined. *n* = 6 mice for control groups, ML-SA1 treatment alone groups, single-dose U exposure alone groups and single-dose U exposure plus ML-SA1 treatment groups. *n* = 4 mice for multiple-dose U exposure alone group and multiple-dose U exposure plus ML-SA1 treatment group. Images share the same scale bar (20 μm) in **a** and the last two lines of **b**. Scale bar of images in the first line of **b** is 100 μm.

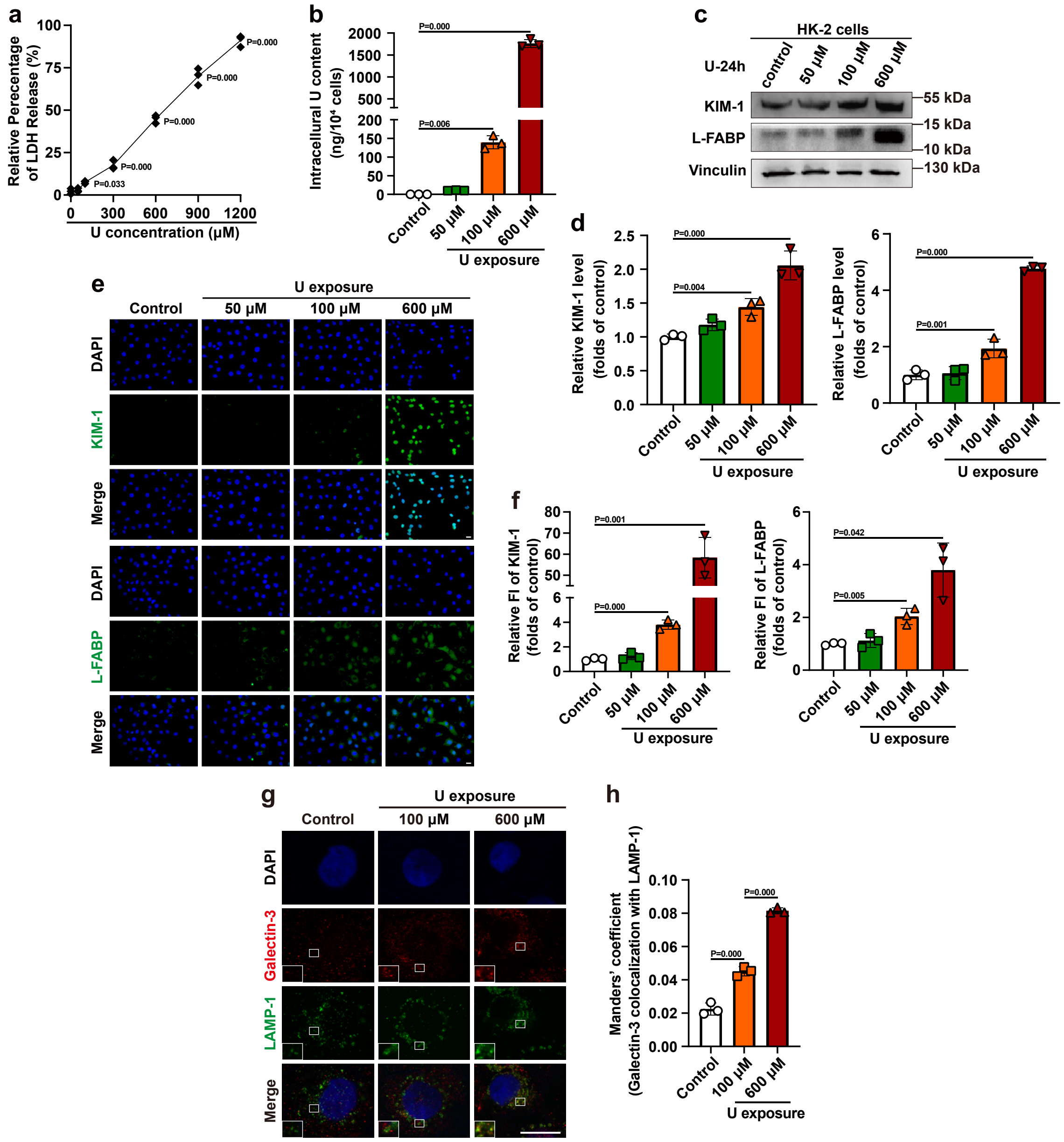

a
Relative Perecentage of LDH Release (%)
U concentration (μM)
P=0.033
P=0.000
b
Intracellural U content (ng/10⁴ cells)
P=0.000
P=0.006
Control
50 μM
100 μM
600 μM
U exposure
c
HK-2 cells
U-24h
control
KIM-1
L-FABP
Vinculin
55 kDa
15 kDa
10 kDa
130 kDa
d
Relative KIM-1 level (folds of control)
P=0.004
Relative L-FABP level (folds of control)
P=0.001
e
DAPI
KIM-1
Merge
L-FABP
f
Relative FI of KIM-1 (folds of control)
Relative FI of L-FABP (folds of control)
P=0.042
P=0.005
g
Galectin-3
LAMP-1
h
Manders' coefficient (Galectin-3 colocalization with LAMP-1)

Supplementary Figure 7. U exposure induces the cytotoxicity, U accumulation and LMP in renal epithelial HK-2 cells. (**a**) U-induced cytotoxicity in HK-2 cells assessed by LDH assay after exposure to U at 0, 50, 100, 300, 600, 900, 1200 μM for 24 h. (**b**) Intracellular U content in HK-2 cells after exposures to U at 0, 50, 100, 600 μM for 24 h. (**c**) Representative western blotting analysis of KIM-1 and L-FABP in HK-2 cells after U exposure at 0, 50, 100, 600 μM for 24 h. (**d**) Quantitative analysis of protein levels of KIM-1 and L-FABP as shown in **c**. (**e**) Representative images of immunofluorescence staining of KIM-1 (green) and L-FABP (green) in HK-2 cells after exposures to U at 0, 50, 100, 600 μM for 24 h. (**f**) Quantitative analysis of KIM-1 and L-FABP staining as shown in **e.** More than 500 cells were analyzed in each sample. (**g**) Representative fluorescence images of colocalization of galectin-3 (red) and LAMP-1 (green) in HK-2 cells after exposures to U at 0, 100, 600 μM for 24 h. (**h**) Quantitative analysis of co-localization of galectin-3 and LAMP-1 as shown in **g**. Thirty cells were analyzed in each sample. Data represent mean ± SD. n = 3 independent experiments. Statistical significance was evaluated by one-way ANOVA with LSD's post hoc test in **a**, **b**, **d**, **h** or two-tailed Student's t-test in **f**. Source data are provided as a Source Data file. All the images share the same scale bar (20 μm).

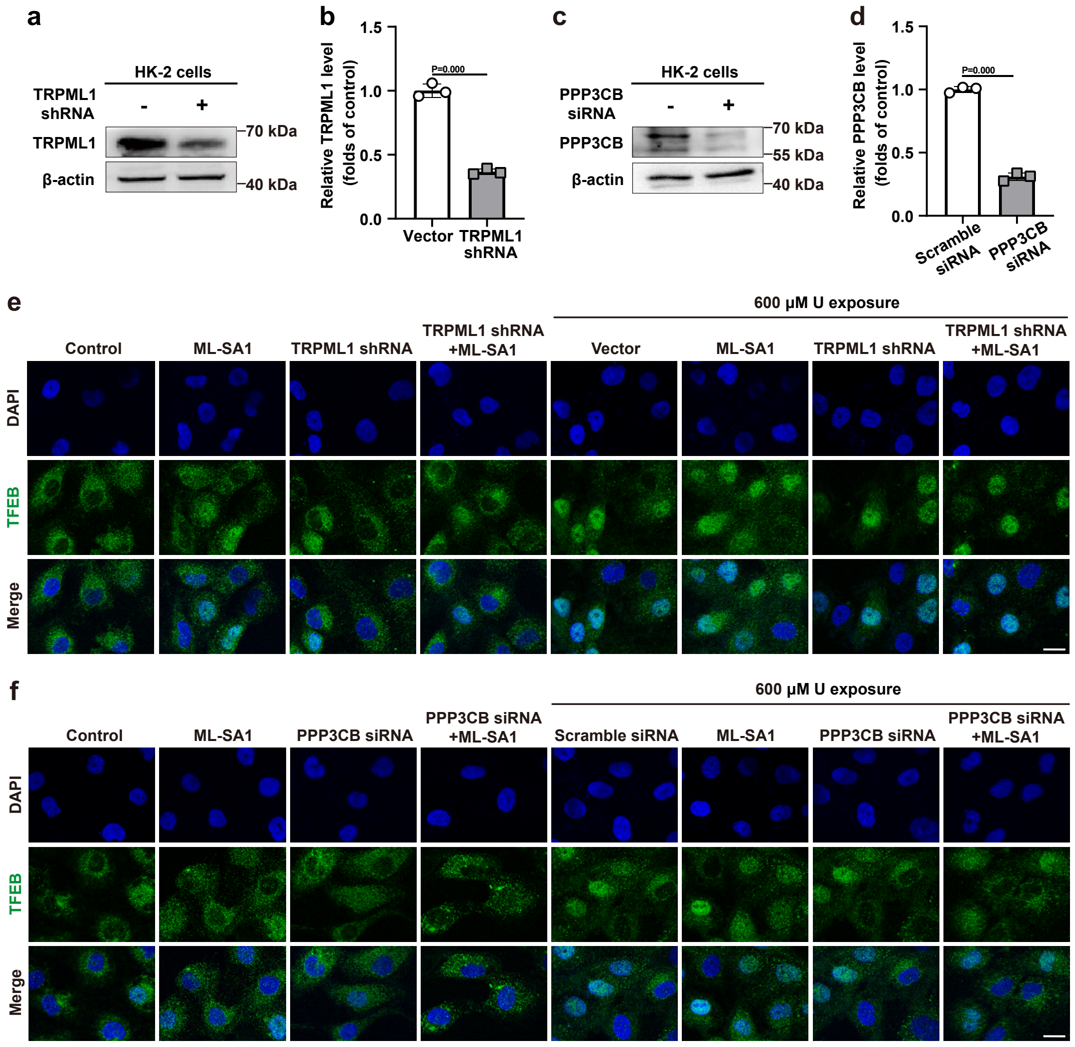
a
HK-2 cells
TRPML1 shRNA
-
+
TRPML1
β-actin
70 kDa
40 kDa
b
Relative TRPML1 level (folds of control)
P=0.000
1.5
1.0
0.5
0.0
Vector
TRPML1 shRNA
c
HK-2 cells
PPP3CB siRNA
-
+
PPP3CB
β-actin
70 kDa
55 kDa
40 kDa
d
Relative PPP3CB level (folds of control)
P=0.000
1.5
1.0
0.5
0.0
Scramble siRNA
PPP3CB siRNA
e
600 μM U exposure
Control
ML-SA1
TRPML1 shRNA
TRPML1 shRNA +ML-SA1
Vector
ML-SA1
TRPML1 shRNA
TRPML1 shRNA +ML-SA1
DAPI
TFEB
Merge
f
600 μM U exposure
Control
ML-SA1
PPP3CB siRNA
PPP3CB siRNA +ML-SA1
Scramble siRNA
ML-SA1
PPP3CB siRNA
PPP3CB siRNA +ML-SA1
DAPI
TFEB
Merge

Supplementary Figure 8. Knockdown of TRPML1 or PPP3CB abrogates the effects of ML-SA1 on the increase of U-induced TFEB nuclear translocation in U-loaded renal epithelial HK-2 cells. HK-2 cells transfected with TRPML1 shRNA, PPP3CB siRNA or corresponding empty vector plasmid/scramble control siRNA were exposed to U at 0, 100 and 600 μM for 24 h. After washout of U, the cells were treated with vehicle or ML-SA1 at 10 μM for 30 min and then analyzed. (**a, b**) Western blotting analysis of TRPML1 levels in HK-2 cells transfected with empty vector or TRPML1 shRNA plasmid. (**c, d**) Western blotting analysis of PPP3CB levels in HK-2 cells transfected with scramble control siRNA or PPP3CB siRNA. **(e, f)** Representative images of immunofluorescence staining of TFEB (green) in TRPML1- or PPP3CB-knockdown HK-2 cells after U exposure and ML-SA1 treatment as indicated in the figure. Data represent mean ± SD. *n* = 3 independent experiments. Statistical significance was evaluated by two-tailed Student's t-test in **b**, **d**. Source data are provided as a Source Data file. All the images share the same scale bar (20 μm).

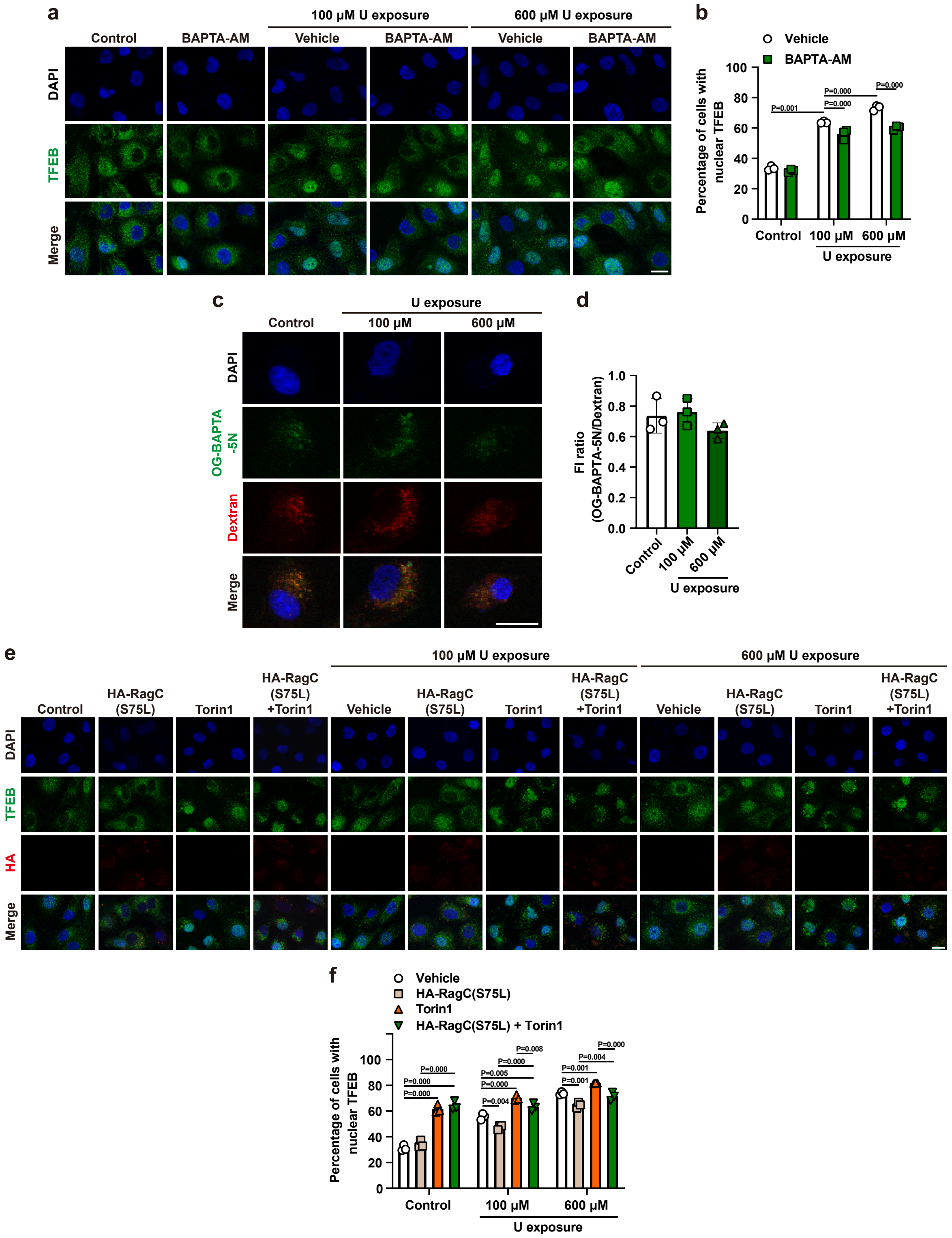

a
100 μM U exposure
600 μM U exposure
Control
BAPTA-AM
Vehicle
BAPTA-AM
Vehicle
BAPTA-AM
DAPI
TFEB
Merge
b
Vehicle
BAPTA-AM
Percentage of cells with nuclear TFEB
P=0.001
P=0.000
P=0.000
P=0.000
Control
100 μM
600 μM
U exposure
c
U exposure
Control
100 μM
600 μM
DAPI
OG-BAPTA -5N
Dextran
Merge
d
FI ratio (OG-BAPTA-5N/Dextran)
Control
100 μM
600 μM
U exposure
e
100 μM U exposure
600 μM U exposure
Control
HA-RagC (S75L)
Torin1
HA-RagC (S75L) +Torin1
Vehicle
HA-RagC (S75L)
Torin1
HA-RagC (S75L) +Torin1
Vehicle
HA-RagC (S75L)
Torin1
HA-RagC (S75L) +Torin1
DAPI
TFEB
HA
Merge
f
Vehicle
HA-RagC(S75L)
Torin1
HA-RagC(S75L) + Torin1
Percentage of cells with nuclear TFEB
P=0.000
P=0.000
P=0.000
P=0.008
P=0.000
P=0.005
P=0.000
P=0.004
P=0.000
P=0.004
P=0.001
P=0.001
Control
100 μM
600 μM
U exposure

Supplementary Figure 9. U exposure-induced TFEB nuclear translocation is dependent on Ca^{2+} efflux from lysosomes and inhibited by active RagC in renal epithelial HK-2 cells. **(a)** Representative images of immunofluorescence staining of TFEB in control and U-loaded HK-2 cells incubated with 100 and 600 μM U for 24 h followed by the treatment of the Ca^{2+} chelators BAPTA-AM (25 μM) for 15 min in nominally Ca^{2+}-free medium containing 1 mM EGTA. **(b)** Quantification of nuclear TFEB shown in **a**. More than 200 cells were analyzed in each sample. **(c)** Representative fluorescence images of lysosomal Ca^{2+} staining in control and U-loaded HK-2 cells incubated with 100 and 600 μM U for 24 h, as indicated by the OG-BAPTA-5N staining in dextran-positive puncta. **(d)** Quantification of lysosomal Ca^{2+} levels shown in **c**. Thirty cells were analyzed in each sample. (**e**) Representative images of immunofluorescence staining of TFEB in HA-RagC(S75L)-overexpression HK-2 cells and Torin1-treated HK-2 cells after U exposure as indicated in the figure. (**f**) Quantification of nuclear TFEB shown in **e.** More than 200 cells were analyzed in each sample. FI: fluorescence intensity. All the images share the same scale bar (20 μm). Data represent mean ± SD. $n = 3$ independent experiments. Statistical significance was evaluated by one-way ANOVA with LSD's post hoc test in **b**, **d**, **f**. Source data are provided as a Source Data file.

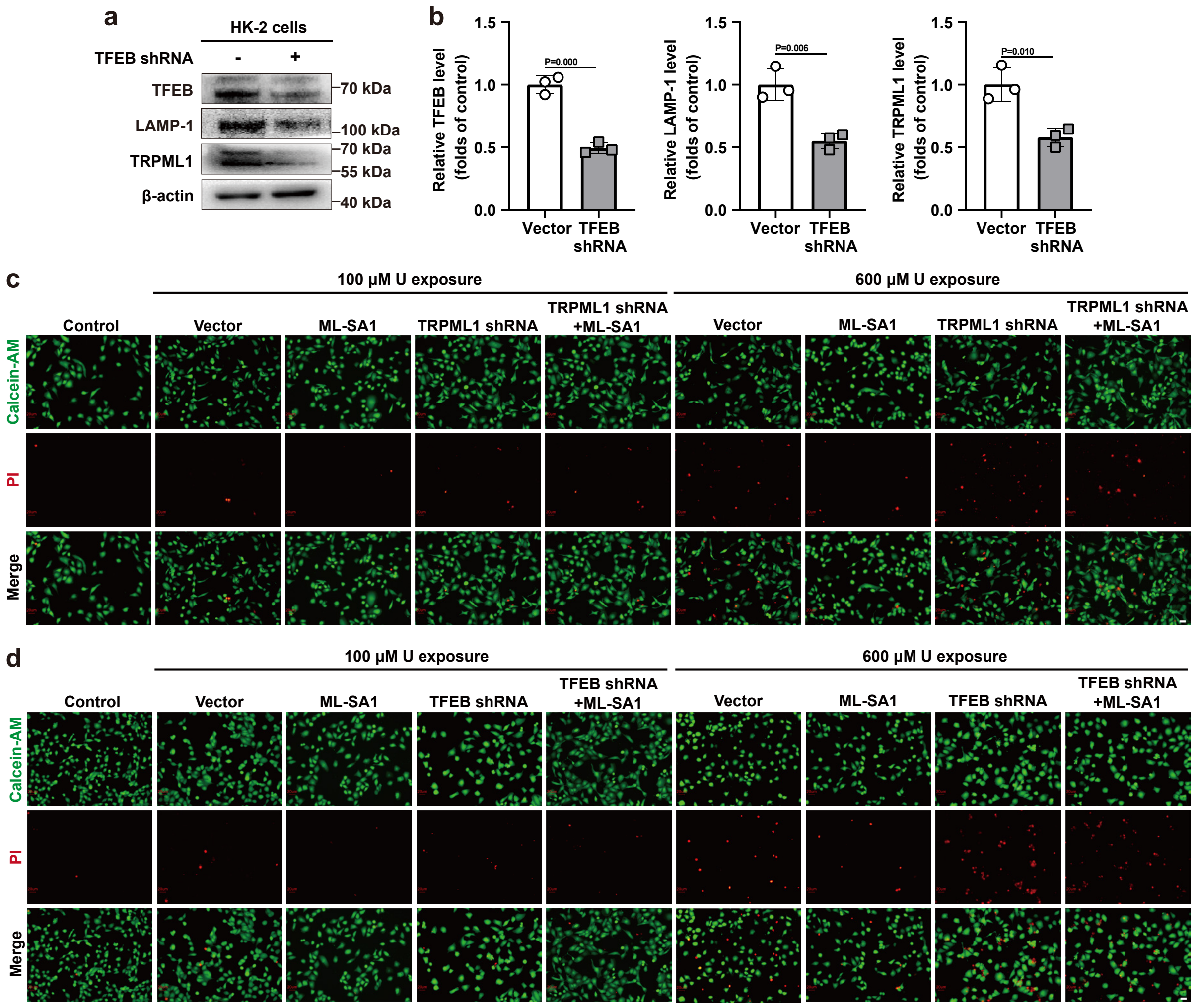

a
HK-2 cells
TFEB shRNA
-
+
TFEB
LAMP-1
TRPML1
β-actin
70 kDa
100 kDa
70 kDa
55 kDa
40 kDa
b
Relative TFEB level (folds of control)
Relative LAMP-1 level (folds of control)
Relative TRPML1 level (folds of control)
P=0.000
P=0.006
P=0.010
1.5
1.0
0.5
0.0
Vector
TFEB shRNA
c
100 μM U exposure
600 μM U exposure
Control
Vector
ML-SA1
TRPML1 shRNA
TRPML1 shRNA +ML-SA1
Calcein-AM
PI
Merge
d
100 μM U exposure
600 μM U exposure
Control
Vector
ML-SA1
TFEB shRNA
TFEB shRNA +ML-SA1
Calcein-AM
PI
Merge

Supplementary Figure 10. Knockdown of TRPML1 or TFEB abolishes the effects of ML-SA1 on the reduction of U-induced cell death in U-loaded renal epithelial HK-2 cells. HK-2 cells transfected with TRPML1 shRNA, TFEB shRNA or corresponding empty vector plasmids were exposed to U at 0, 100 and 600 μM for 24 h. After washout of U, the cells were treated with vehicle or ML-SA1 at 10 μM for 30 min and then analyzed. (**a, b**) Western blotting analysis of TFEB, TRPML1 and LAMP-1 levels in HK-2 cells transfected with empty vector or TFEB shRNA plasmid. Data represent mean ± SD. $n = 3$ independent experiments. Statistical significance was evaluated by two-tailed Student's t-test in **b**. Source data are provided as a Source Data file. (**c**) Representative fluorescence images of Calcein-AM/PI staining in HK-2 cells transfected with empty vector or TRPML1 shRNA plasmid after U exposure and ML-SA1 treatment. $n = 3$ independent experiments. (**d**) Representative fluorescence images of Calcein-AM/PI staining in HK-2 cells transfected with empty vector or TFEB shRNA plasmid after U exposure and ML-SA1 treatment. The living cells were stained with Calcein-AM (green) and the nucleus of the dead cells were stained with PI (red). $n = 3$ independent experiments. All the images share the same scale bar (20 μm).

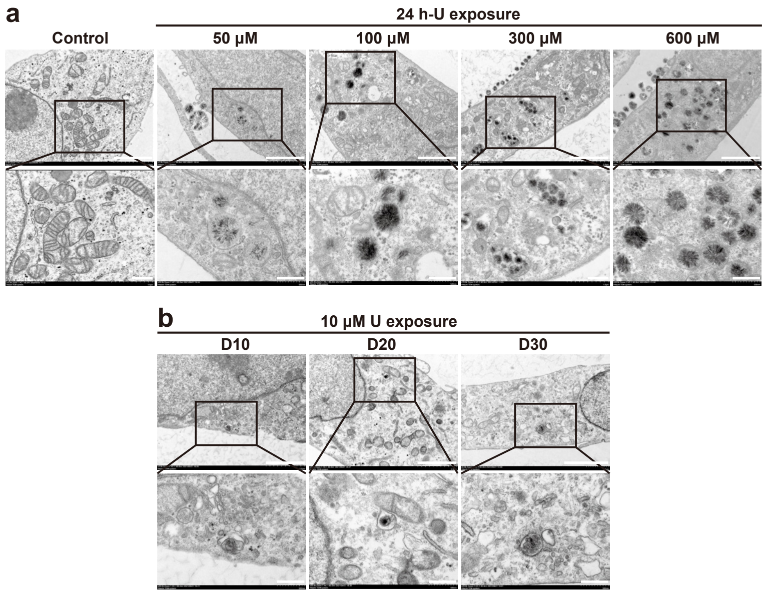
a
24 h-U exposure
Control
50 μM
100 μM
300 μM
600 μM
b
10 μM U exposure
D10
D20
D30

Supplementary Figure 11. U precipitates with needle-like structure are distributed in lysosome-like vesicles in U-loaded renal epithelial HK-2 cells. (**a**) TEM images of HK-2 cells exposed to U at 0, 50, 100, 300, 600 μM for 24 h. Boxed areas are enlarged below. The needle-shaped structures of U precipitates in lysosomes, autolysosomes and multivesicular bodies within the cells after 24 h exposure to U at 50, 100, 300 and 600 μM. (**b**) TEM images of HK-2 cells exposed to U at 10 μM for 10, 20, 30 days. Boxed areas are enlarged below. No precipitates were observed within the cells. $n = 2$ independent experiments. Images share the same scale bar (2 μm) in the first line of **a** and **b**. Scale bar of images in the second line of **a** and **b** is 500 nm.

Supplementary Table 1. U content in the kidney of U-exposed mice with perfusion and without perfusion.

Groups	No. of mice	U dosage	U content in the kidney (μg/g tissue)[c]	
			Perfusion	Without perfusion
A single dose of U-exposed group[a]	5	0.4 mg/kg	4.64 ± 0.66	4.66 ± 1.17
A single dose of U-exposed group[a]	5	2.0 mg/kg	25.71 ± 8.80	23.55 ± 4.91
multiple doses of U-exposed group[b]	6	80 μg/kg/d*5 d	2.96 ± 0.44	2.88 ± 0.33

[a]Mice were irrigated 48 h after intramuscular injection of single-dose U.

[b]Mice were irrigated 48 h after the last intramuscular injection of multiple-doses U.

[c]Data represent mean ± SD.

Supplementary Materials and Methods

Drug, reagent and plasmid. Sodium bicarbonate was purchased from Shanghai Yurui Biotechnology (Anyang) pharmaceutical Co., LTD (China). Sodium bicarbonate was dissolved in distilled water, and administrated at 12.5 mL/kg body weight. Torin 1 (#S2827) and BAPTA-AM (#S7534) were from Selleck. Plasmid pRK5-HA GST RagC 75L (#19305) was obtained from Addgene[1].

Antibodes. Both anti-KIM-1 (LifeSpan BioSciences, #LS-B2103) and anti-L-FABP (HuaBio, #EM170403) were diluted 1:1000 for western blotting and immunofluorescence staining. Anti-PPP3CB (Absin, China, #abs111623) and anti-TFEB (Beyotime Biotechnology, #AF8103) were diluted 1:1000 for western blotting. Anti-HA-Tag (Cell Signaling Technology, #2367) was diluted 1:200 for immunofluorescence staining.

Animal experiments. Male Balb/c mice at 7 weeks of age were randomly assigned to each group (three animals per group). For experiment I, there were four groups of mice and all the mice in groups 1-3 were injected intramuscularly with a single dose of U (2.0 mg/kg body weight) into the right femoral muscle. The mice in group 1 were then given a single oral administration of sodium bicarbonate at the dose of 1 g/kg 10 min after the U injection. The dosage of sodium bicarbonate was based on the study by Ohmachi et al[2]. The mice in group 2 were given a single oral administration of sodium bicarbonate at the dose of 1 g/kg 24 h after the U injection. The mice in group 3 were given a single oral administration of normal saline 10 min after the U injection. The mice in the blank control group (group 4) were intramuscularly

and orally administered by normal saline. For experiment II, there were three groups of mice, and all the mice in groups 1 & 2 were injected intramuscularly into the right femoral muscle with multiple doses of U at 80 μg/kg once daily for 5 days. Twenty-four hours after the last U injection, the mice in group 1 and 2 were given a single oral administration of sodium bicarbonate at a dose of 1 g/ kg and normal saline, respectively. The mice in the blank control group (group 3) were intramuscularly and orally administered by normal saline in the same scheme. All the mice were housed singly in plastic metabolism cages to collect urine every 24 h and the pH of urine was detected by chemical urinalysis strips. At 48 h after the single U injection in experiment I or last U injection for repeated U injection in experiment II, the mice were anesthetized and sacrificed. Blood samples were collected from the orbital venous sinus in the mice and the serum was separated by centrifugation at 3000 rpm for 5 min. The kidneys from both sides were obtained after perfusion with PBS at 4°C. Urine and one kidney from each mouse were collected for U content detection, and the other kidney was for paraffin embedding section.

Cytotoxicity assays. HK-2 cells in 96-well plates were exposed to U at 0, 50, 100, 300, 600, 900 and 1200 μM for 24 h. The U-induced cytotoxicity was measured using the lactate dehydrogenase (LDH) assay kit (Beyotime Biotechnology, China, #C0016) according to the manufacturer's instructions.

Lysosomal [Ca^{2+}] Measurement. Lysosomal Ca^{2+} was measured by fluorescence ratio imaging of an Oregon Green 488 BAPTA-5N and Texas Red-conjugated dextran (10000 MW) as

previously described[3,4] with minor modifications. Briefly, HK-2 cells were loaded with membrane-impermeant Oregon Green 488 BAPTA-5N (a pH-insensitive Ca^{2+} indicator, 10 μM) (Thermo Fisher Scientific, #O6812) and Texas Red-conjugated dextran (Ca^{2+} insensitive, 0.5 mg/ml) (Thermo Fisher Scientific, #D1863) for 24 h to induce lysosomal accumulation of dyes through endocytosis, and simultaneously exposed to U at 0, 100 and 600 μM for 24 h. Images were taken using a Leica SP8 confocal laser scanning microscope (Germany), and the fluorescent intensity of Oregon Green 488 BAPTA-5N was normalized to the fluorescence intensity of Texas Red-conjugated dextran in endocytic vesicles of cells using Image J 1.8.0 software (National Institute of Health, USA), and the normalized fluorescent intensity of Oregon Green 488 BAPTA-5N is indicative of the luminal Ca^{2+} concentration in lysosomes.

Transmission electron microscopy. After rinsing with PBS containing 10 mM sodium bicarbonate, HK-2 cells in 100 mm dishes were *in situ* fixed with 2.5% glutaraldehyde fixative solution at room temperature for 5 min. The cells were then gently scraped in one direction using a cell scraper and sucked into a 15 ml centrifuge tube. Cell pellets were collected by centrifugation at 1158 g for 2 min, fixed with 2.5% glutaraldehyde fixative solution at room temperature for 30 min, and stored at 4°C for transporting to Wuhan Servicebio Technology Co., Ltd (China) for TEM. Briefly, the fixed cells were washed with 0.1 M PBS (pH 7.4), pre-embedded with 1% agarose solution, postfixed with 1% osmium tetroxide (OsO4, Servicebio) for 2 h, rinsed with 0.1 M PBS (pH 7.4), dehydrated in a graded series of ethanol (30, 50, 70, 80, 95, and 100%), and embedded in EMBed 812 resin. The resin was polymerized at 65°C for more than 48 h, and cut into ultrathin sections (60-80 nm thick). The ultrathin sections were put

on copper grids, stained with 2% uranyl acetate (Servicebio) and 2.6% lead citrate (Servicebio) and examined and photographed with a transmission electron microscopy (HT7700, HITACHI, Japan).

Supplementary References

1. Sancak, Y.*, et al.* The Rag GTPases bind raptor and mediate amino acid signaling to mTORC1. *Science* **320**, 1496-1501 (2008).
2. Ohmachi, Y.*, et al.* Sodium bicarbonate protects uranium-induced acute nephrotoxicity through uranium-decorporation by urinary alkalinization in rats. *J Toxicol Pathol* **28**, 65-71 (2015).
3. Cao, Q.*, et al.* BK Channels Alleviate Lysosomal Storage Diseases by Providing Positive Feedback Regulation of Lysosomal Ca^{2+} Release. *Dev Cell* **33**, 427-441 (2015).
4. Nakamura, S.*, et al.* LC3 lipidation is essential for TFEB activation during the lysosomal damage response to kidney injury. *Nat Cell Biol* **22**, 1252-1263 (2020).
