# Supplementary material for: Induction of lysosomal exocytosis and biogenesis via TRPML1 activation for the treatment of uranium-induced nephrotoxicity
Source: Nat Commun. 2023 Jul 6;14:3997. doi: 10.1038/s41467-023-39716-7 (PMC10326073; doi:10.1038/s41467-023-39716-7)
Supplement: Supplementary file 3 — Reporting Summary [file 41467_2023_39716_MOESM3_ESM.pdf]

nature portfolio

Corresponding author(s): Honghong Chen

Last updated by author(s): Jun 19, 2023

Reporting Summary

Nature Portfolio wishes to improve the reproducibility of the work that we publish. This form provides structure for consistency and transparency in reporting. For further information on Nature Portfolio policies, see our Editorial Policies and the Editorial Policy Checklist.

Statistics

For all statistical analyses, confirm that the following items are present in the figure legend, table legend, main text, or Methods section.

n/a	Confirmed	
☐	☒	The exact sample size (n) for each experimental group/condition, given as a discrete number and unit of measurement
☐	☒	A statement on whether measurements were taken from distinct samples or whether the same sample was measured repeatedly
☐	☒	The statistical test(s) used AND whether they are one- or two-sided<br>*Only common tests should be described solely by name; describe more complex techniques in the Methods section.*
☒	☐	A description of all covariates tested
☒	☐	A description of any assumptions or corrections, such as tests of normality and adjustment for multiple comparisons
☐	☒	A full description of the statistical parameters including central tendency (e.g. means) or other basic estimates (e.g. regression coefficient) AND variation (e.g. standard deviation) or associated estimates of uncertainty (e.g. confidence intervals)
☐	☒	For null hypothesis testing, the test statistic (e.g. F, t, r) with confidence intervals, effect sizes, degrees of freedom and P value noted<br>*Give P values as exact values whenever suitable.*
☒	☐	For Bayesian analysis, information on the choice of priors and Markov chain Monte Carlo settings
☒	☐	For hierarchical and complex designs, identification of the appropriate level for tests and full reporting of outcomes
☒	☐	Estimates of effect sizes (e.g. Cohen's d, Pearson's r), indicating how they were calculated

Our web collection on statistics for biologists contains articles on many of the points above.

Software and code

Policy information about availability of computer code

Data collection	ICP-MS data: NexIon (PekinElmer, version 1.5).<br>Microscopic data: ZEISS microscope (Imager M2, ZEISS) operated by ZEN Blue Imaging Analysis (ZEISS, version 2016.08.06), Leica SP8 confocal laser scanning microscope operated by Leica Application Suite X (Leica, version 3.5.6.21594), Nikon microscope (ECLIPSE Ts2-FL, Nikon) operated by CapStudio software (iMG, version biology 3.7.2). ImageXpress Micro 4 screening system (Molecular Devices) operated by MetaXpress software (Molecular Devices, version 6.6.1.42).<br>Ca2+ imaging data: FLIPR Penta high-throughput real-time fluorescence imaging analysis system (FLIPR) operated by FLIPR Penta (Molecular Devices).<br>Immunoblotting data: ChemiDoc XRS system (Bio-Rad).
Data analysis	Statistical analysis: IBM SPSS Statistics 20.0 (IBM SPSS).<br>Immunohistochemistry staining and immunofluorescence staining analysis: ImageJ 1.8.0 software (NIH).

For manuscripts utilizing custom algorithms or software that are central to the research but not yet described in published literature, software must be made available to editors and reviewers. We strongly encourage code deposition in a community repository (e.g. GitHub). See the Nature Portfolio guidelines for submitting code & software for further information.

Data

Policy information about availability of data

All manuscripts must include a data availability statement. This statement should provide the following information, where applicable:

- Accession codes, unique identifiers, or web links for publicly available datasets
- A description of any restrictions on data availability
- For clinical datasets or third party data, please ensure that the statement adheres to our policy

All data supporting the findings of this work are available within the paper and its Supplementary information files. Source data are provided with this paper.

Human research participants

Policy information about studies involving human research participants and Sex and Gender in Research.

Reporting on sex and gender	This study did not involve human research participants.
Population characteristics	This study did not involve human research participants.
Recruitment	This study did not involve human research participants.
Ethics oversight	This study did not involve human research participants.

Note that full information on the approval of the study protocol must also be provided in the manuscript.

Field-specific reporting

Please select the one below that is the best fit for your research. If you are not sure, read the appropriate sections before making your selection.

☒ Life sciences ☐ Behavioural & social sciences ☐ Ecological, evolutionary & environmental sciences

For a reference copy of the document with all sections, see nature.com/documents/nr-reporting-summary-flat.pdf

Life sciences study design

All studies must disclose on these points even when the disclosure is negative.

Sample size	We followed the routine practice based on the need to have sufficient statistical power in the same study field (PMID: 31239437, 23454449, 20699704, 18849679, 15951694, 10772024), such that no sample size calculation was performed.
Data exclusions	No data were excluded from the analysis.
Replication	All experiments were repeated independently at least 3 times to ensure reproducibility of the experimental outcomes as detailed in the figure legends.
Randomization	Mice were randomized to receive treatment as described. Images related in all experiments were acquired randomly. Randomization was not relevant for cell culture study since the starting material (i.e. HK-2 cells) was the same for all conditions and subsequently underwent different experimental treatment.
Blinding	The investigators were blinded to all grouping information in data collection and analysis of each experiment.

Reporting for specific materials, systems and methods

We require information from authors about some types of materials, experimental systems and methods used in many studies. Here, indicate whether each material, system or method listed is relevant to your study. If you are not sure if a list item applies to your research, read the appropriate section before selecting a response.

Materials & experimental systems

n/a	Involved in the study
☐	☒ Antibodies
☐	☒ Eukaryotic cell lines
☒	☐ Palaeontology and archaeology
☐	☒ Animals and other organisms
☒	☐ Clinical data
☒	☐ Dual use research of concern

Methods

n/a	Involved in the study
☒	☐ ChIP-seq
☒	☐ Flow cytometry
☒	☐ MRI-based neuroimaging

Antibodies

Antibodies used

Primary antibodies used for immunohistochemical staining were as follows:
anti-KIM-1 (LifeSpan BioSciences, Cat#LS-B2103-50, Lot#72775, Dilution: 1:400)
anti-LAMP-1 (Invitrogen, Cat#14-1071-82, Clone: eBio1D4B (1D4B), Lot#2218070, Dilution: 1:200)
anti-TRPML-1 (Atlas Antibodies, Cat#: HPA031763, Lot#000022744, Dilution: 1:200)
anti-Galectin-1 (HuaBio, Cat#ET1705-83, Clone: JM13-37, Lot#H01008, Dilution: 1:200)

Primary antibodies used for immunofluorescence staining were as follows:
anti-LAMP-1 (Cell Signaling Technology, Cat#9091, Clone: D2D11, Lot#7, Dilution: 1:200)
anti-TRPML-1 (Atlas Antibodies, Cat#: HPA031763, Lot#000022744, Dilution: 1:200)
anti-Galectin-3 (BD Biosciences, Cat#556904, Clone: B2C10, Lot#9049555, Dilution: 1:200)
anti-cleaved-caspase-3 (Cell Signaling Technology, Cat#9664, Clone: 5A1E, Lot#22, Dilution: 1:400)
anti-TFEB (Cell Signaling Technology, Cat#4240, Lot#3, Dilution: 1:200)
anti-KIM-1 (LifeSpan BioSciences, Cat#LS-B2103-50, Lot#72775, Dilution: 1:1000)
anti-L-FABP (HuaBio, Cat#EM170403, Clone: AH54-31, Lot#HK1201, Dilution: 1:1000)
anti-HA-Tag (Cell Signaling Technology, Cat#2367, Clone: 6E2, Lot#5, Dilution: 1:200)

Primary antibodies used for Western blotting were as follows:
anti-TFEB (Cell Signaling Technology, Cat#4240, Lot#3, Dilution: 1:1000)
anti-β-Actin (Cell Signaling Technology, Cat#4970, Clone: 13E5, Lot#5, Dilution: 1:1000)
anti-LAMP-1 (Cell Signaling Technology, Cat#9091, Clone: D2D11, Lot#7, Dilution: 1:1000)
anti-TRPML-1 (Atlas Antibodies, Cat#: HPA031763, Lot#000022744, Dilution: 1:1000)
anti-phospho-p70 S6 Kinase (Cell Signaling Technology, Cat#9234, Clone: 108D2, Lot#12, Dilution: 1:1000)
anti-p70 S6 Kinase (Cell Signaling Technology, Cat#9202, Lot#20, Dilution: 1:1000)
anti-KIM-1 (LifeSpan BioSciences, Cat#LS-B2103-50, Lot#72775, Dilution: 1:1000)
anti-L-FABP (HuaBio, Cat#EM170403, Clone: AH54-31, Lot#HK1201, Dilution: 1:1000)
anti-Vinculin (Cell Signaling Technology, Cat#4650, Lot#4, Dilution: 1:1000)
anti-PPP3CB (Absin, Cat#abs111623, Lot#628A005, Dilution: 1:1000)
anti-TFEB (Beyotime Biotechnology, Cat#AF8130, Dilution: 1:1000)

Secondary antibodies used for immunohistochemical staining were as follows:
HRP-polymer-conjugated secondary antibody (Proteintech, Cat#PK10006, No dilution)

Secondary antibodies used for immunofluorescence staining were as follows:
Donkey anti-Rabbit IgG (H+L) Highly Cross-Adsorbed Secondary Antibody, Alexa Fluor 555 (Invitrogen, Cat#A31572, Lot#2339822, Dilution: 1:500)
Donkey anti-Mouse IgG (H+L) Highly Cross-Adsorbed Secondary Antibody, Alexa Fluor 555 (Invitrogen, Cat#A31570, Lot#2045336, Dilution: 1:500)
Donkey anti-Rabbit IgG (H+L) Highly Cross-Adsorbed Secondary Antibody, Alexa Fluor 488 (Invitrogen, Cat#A21206, Lot#2376850, Dilution: 1:500)
Donkey anti-Mouse IgG (H+L) Highly Cross-Adsorbed Secondary Antibody, Alexa Fluor 488 (Invitrogen, Cat#A21202, Lot#1915874, Dilution: 1:500)

Secondary antibodies used for Western blotting were as follows:
HRP-conjugated goat anti-rabbit IgG (Beyotime Biotechnology, Cat#A0208, Dilution: 1:1000)
HRP Conjugated Goat anti-Mouse IgG Goat Polyclonal Antibody (HuaBio, Cat#HA1006, Lot# G160529, Dilution: 1:1000)

Validation

Commercial antibodies used in this study were validated by the manufacturer as reported in the antibody data sheet.

anti-KIM-1 (https://www.lsbio.com/pathplus-antibodies/pathplus-havcr1-antibody-kim-1-antibody-elisa-if-immunofluorescence-ihc-wb-western-ls-b2103/56177)

anti-LAMP-1 (https://www.thermofisher.cn/cn/zh/antibody/product/CD107a-LAMP-1-Antibody-clone-eBio1D4B-1D4B-Monoclonal/14-1071-82)

anti-TRPML-1 (https://www.atlasantibodies.com/products/antibodies/primary-antibodies/triple-a-polyclonals/mcoln1-antibody-hpa031763)

anti-Galectin-1 (http://www.huabio.cn/product/Galectin-1-antibody-ET1705-83)

anti-LAMP-1 (https://www.cellsignal.cn/products/primary-antibodies/lamp1-d2d11-xp-rabbit-mab/9091)

anti-Galectin-3 (https://www.bdbiosciences.com/zh-cn/products/reagents/flow-cytometry-reagents/research-reagents/single-color-antibodies-ruo/purified-mouse-anti-human-galectin-3.556904)

anti-cleaved-caspase-3(https://www.cellsignal.cn/products/primary-antibodies/cleaved-caspase-3-asp175-5a1e-rabbit-mab/9664)

anti-TFEB (https://www.cellsignal.cn/products/primary-antibodies/tfeb-antibody/4240)

anti-L-FABP (http://www.huabio.cn/product/L-FABP-antibody-EM170403)

anti-HA-Tag (https://www.cellsignal.cn/products/primary-antibodies/ha-tag-6e2-mouse-mab/2367)

anti-β-Actin (https://www.cellsignal.cn/products/primary-antibodies/b-actin-13e5-rabbit-mab/4970)

anti-phospho-p70 S6 Kinase (https://www.cellsignal.cn/products/primary-antibodies/phospho-p70-s6-kinase-thr389-108d2-rabbit-mab/9234)

anti-p70 S6 Kinase (https://www.cellsignal.cn/products/primary-antibodies/p70-s6-kinase-antibody/9202)

anti-Vinculin (https://www.cellsignal.cn/products/primary-antibodies/vinculin-antibody/4650)

anti-PPP3CB (https://www.absin.cn/rabbit-ppp3cb-polyclonal-antibody/abs111623.html)

anti-TFEB (https://www.beyotime.com/product/AF8130.htm)

HRP-polymer-conjugated secondary antibody (https://www.ptgcn.com/products/IHC-Detect-Kit-for-Rabbit-Mouse-Primary-Antibody-PK10006.htm)

Donkey anti-Rabbit IgG (H+L) Highly Cross-Adsorbed Secondary Antibody, Alexa Fluor 555 (https://www.thermofisher.cn/cn/zh/antibody/product/Donkey-anti-Rabbit-IgG-H-L-Highly-Cross-Adsorbed-Secondary-Antibody-Polyclonal/A-31572)

Donkey anti-Rabbit IgG (H+L) Highly Cross-Adsorbed Secondary Antibody, Alexa Fluor 488 (https://www.thermofisher.cn/cn/zh/antibody/product/Donkey-anti-Rabbit-IgG-H-L-Highly-Cross-Adsorbed-Secondary-Antibody-Polyclonal/A-21206)

Donkey anti-Mouse IgG (H+L) Highly Cross-Adsorbed Secondary Antibody, Alexa Fluor 555 (https://www.thermofisher.cn/cn/zh/antibody/product/Donkey-anti-Mouse-IgG-H-L-Highly-Cross-Adsorbed-Secondary-Antibody-Polyclonal/A-31570)

Donkey anti-Mouse IgG (H+L) Highly Cross-Adsorbed Secondary Antibody, Alexa Fluor 488 (https://www.thermofisher.cn/cn/zh/antibody/product/Donkey-anti-Mouse-IgG-H-L-Highly-Cross-Adsorbed-Secondary-Antibody-Polyclonal/A-21202)

HRP-conjugated goat anti-rabbit IgG (https://www.beyotime.com/product/A0208.htm)

HRP Conjugated Goat anti-Mouse IgG Goat Polyclonal Antibody (http://www.huabio.cn/product/Goat-anti-Mouse-IgG-HRP-antibody-HA1006)

Eukaryotic cell lines

Policy information about cell lines and Sex and Gender in Research

Cell line source(s)	Human renal proximal tubular epithelial cell line HK-2 (#SCSP-511) was obtained from the Type Culture Collection of the Chinese Academy of Sciences, Shanghai, China.
Authentication	The HK-2 cell line was authenticated through short tandem repeat DNA profiling by the supplier.
Mycoplasma contamination	We confirmed there was no mycoplasma contamination.
Commonly misidentified lines (See ICLAC register)	No commonly misidentified cell lines were used in this study.

Animals and other research organisms

Policy information about studies involving animals; ARRIVE guidelines recommended for reporting animal research, and Sex and Gender in Research

Laboratory animals	BALB/c mice (Cleanliness: SPF) were bought from Shanghai Jiesijie Laboratory Animal Technology Co. Ltd. The 7-week-old male mice received a single intramuscular injection of 0.4 mg/kg or 2.0 mg/kg of U, or five times intramuscular injection of 80 µg/kg/d of U followed intraperitoneal injection of ML-SA1 at 400 µg/kg or 800 µg/kg bw or oral administration of sodium bicarbonate at the dose of 1 g/kg at 10 min or 24 h after im injection of U, and were sacrificed after indicated time periods.<br>Mice were maintained in a specific pathogen free (SPF), environmentally suitable barrier system at 20-26 ℃ and 40-70% humidity with a light/dark cycle of 12/12 hours at the Laboratory Animal Center for Drug Evaluation of School of Pharmacy, Fudan University.

	Mice were fed with a gamma-irradiated AIN-93G purified diet formulated for gestating and growing rodents (Wuxi Fanbo Biotechnology Co., Ltd, Jiangsu province, China, #FB-D10012G), and water ad libitum.
Wild animals	No wild animals were used.
Reporting on sex	All mice used in this study were male as it is not expected that sex and gender may affect the effect of TRPML1 agonist ML-SA1 on U-induced nephrotoxicity. Male mice or rats are commonly used in in vivo uranium decorporation experiments in our lab (PMID: 23454449) and other labs (PMID: 26148447, 15951694). Female mice are also usually used in in vivo uranium decorporation experiments in several labs (PMID: 31239437, 20699704, 20699704, 10772024). There was no report on the difference in decorpoation effect of compounds in uranium-exposed mice or rats of different genders.
Field-collected samples	No applicable.
Ethics oversight	All animal experiments were approved by the Animal Research Ethics Committee of School of Pharmacy of Fudan University (No. 2021-01-FYS-CHH-013).

Note that full information on the approval of the study protocol must also be provided in the manuscript.
